# Controlling Multicomponent Condensate Morphology via Additive-Modulated Interactions

**DOI:** 10.1021/jacsau.5c00713

**Published:** 2025-07-30

**Authors:** Jiahui Wang, Arash Nikoubashman, Young C. Kim, Jeetain Mittal

**Affiliations:** † Artie McFerrin Department of Chemical Engineering, 14736 Texas A&M University, College Station, Texas 77843, United States; ‡ Leibniz-Institut für Polymerforschung Dresden e.V., Hohe Straße 6, 01069 Dresden, Germany; § Institut für Theoretische Physik, Technische Universität Dresden, 01069 Dresden, Germany; ∥ Cluster of Excellence Physics of Life, Technische Universität Dresden, 01062 Dresden, Germany; ⊥ Center for Materials Physics and Technology, 41487 Naval Research Laboratory, Washington, District of Columbia 20375, United States; # Department of Chemistry, Texas A&M University, College Station, Texas 77843, United States; ¶ Interdisciplinary Graduate Program in Genetics and Genomics, Texas A&M University, College Station, Texas 77843, United States

**Keywords:** multicomponent condensate, morphological transition, additive, small molecules, homotypic interaction, heterotypic interaction

## Abstract

The morphology of biomolecular condensates plays a critical role in regulating intracellular organization and function by enabling both spatial and temporal control over biochemical processes. Recent studies have shown that small-molecule cosolutes can not only modulate phase separation but also influence condensate morphology. However, the mechanistic understanding of how small molecules regulate condensate structure remains limited. In this study, we employ coarse-grained molecular dynamics simulations to investigate how the morphology of two-component condensates can be modulated through the introduction of additional small-molecule cosolutes. By systematically varying the interaction strengths between the small molecules and the macromolecular components, we observe morphological transitions between, e.g., core–shell and dewetted structures. To rationalize these transitions, we calculate second virial coefficients in the presence of the small molecules, providing a molecular-level framework to capture shifts in effective homotypic and heterotypic interactions. We further investigate the role of stoichiometry between the small molecules and macromolecules, demonstrating that stoichiometry and interaction strength jointly determine the condensate morphology by altering the relative interaction strengths among components. Additionally, we show that fully mixed two-component condensates can undergo transitions to microphase-separated morphologies, such as core–shell or dewetted, upon small molecule introduction. Together, these findings reveal that condensate morphology can be rationally tuned through interaction- and stoichiometry-dependent mechanisms, offering molecular-scale insights into how small-molecule cosolutes modulate condensate structure.

## Graphical abstract

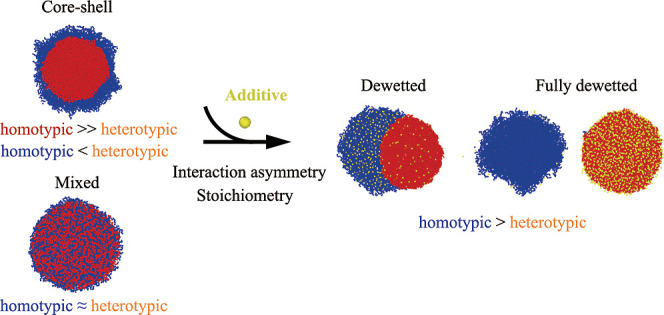

## Introduction

Macromolecular mixtures can undergo phase separation to form multiphase condensates, a phenomenon widely observed in biomolecular condensates.^1–3^ The hierarchical organization of biomolecular condensates has been identified in various cellular structures,^4–7^ such as the multiple phases in the nucleolus^6^ and the core–shell architecture in stress granules,^8^ which are closely associated with condensate properties and functions.^9^ For instance, within the nucleolus, distinct condensate phases organize ribosomal maturation steps in a sequential manner, maintaining structural separation and functional precision to optimize processing efficiency.^6,10^ The ability of biomolecular condensates to form subcompartments enables fine-tuned positional and chronological regulation of complex intracellular processes, drawing increasing scientific attention.

Consequently, research on multiphase condensates has expanded in recent years, exploring their organizational mechanisms from various perspectives. Feric et al. demonstrated that the formation of multiple nucleolar subcompartments is driven by differences in surface tension,^4^ while other studies have also highlighted the role of interfacial tension, an essential mesoscale property, in governing multiphase condensate formation.^11–13^ By investigating the local microenvironments within biomolecular condensates, Ye et al. revealed that local micropolarity differences are crucial for the emergence of multiphase structures.^14^ At the molecular level, Welles/Sojitra et al. proposed that the spatial segregation of condensates results from a balance between homotypic and heterotypic interactions.^15^ Similarly, Rana et al. demonstrated that asymmetric oligomerization among condensate components regulates the miscibility of intrinsically disordered proteins (IDPs), further shaping the phase behavior of biomolecular condensates.^16^

Apart from understanding their formation mechanisms, recent efforts have also focused on how to regulate condensate morphology by modifying system composition, including the introduction of macromolecules,^200–19^ crowding agents,^20,21^ pH variation,^22,23^ and salt concentration.^24,25^ Notably, Zhu et al. reported that the introduction of small-molecule cosolutes, such as ions from the Hofmeister series, can alter condensate microenvironments and reshape condensate morphology.^26^ These findings highlight the emerging importance of small-molecule cosolutes in modulating condensate architecture.

Indeed, small-molecule cosolutes have recently begun to be studied as modulators of biomolecular phase separation.^27,28^ For example, Babinchak et al. showed that Bis-ANS, a fluorescent small molecule, acts as a biphasic modulator of protein condensation, capable of both inducing phase separation and preventing droplet formation.^29^ ATP as a similar biphasic modulator has also been reported.^30–32^ Wang et al. further demonstrated that small molecules such as Ro-3306 can alter the material properties of TFEB condensates, enhancing droplet size and fusion propensity, thereby modulating autophagosome-lysosome dynamics.^33^ These studies highlight the important role of small-molecule cosolutes in biomolecular condensate research, providing powerful tools for uncovering the mechanisms of phase separation and offering promising strategies for therapeutic intervention in condensate-related diseases.

Despite growing evidence of the regulatory role of small molecules in biomolecular condensates, a fundamental understanding of how such small-molecule cosolutes influence the morphology of multicomponent condensates remains limited. Therefore, in this study, we aim to establish a general framework that directly links molecular interaction parameters to the mesoscale phase behavior of macromolecular systems, thereby elucidating the principles underlying morphology control through the introduction of small molecules. Recognizing that biomolecular condensates, typically composed of proteins and nucleic acids, often arise from multivalent interactions that can be effectively captured by simplified macromolecular models,^2,15,34–36^ we employ simulations of a binary macromolecular system with the addition of small molecules. By systematically varying the interaction strengths between the small molecules and the macromolecular components, we investigate the resulting morphology transitions. This approach builds upon previous studies that have successfully utilized comparable models to probe diverse chemical phenomena.^37–39^ For example, Li et al. employed a similar model to explore the fabrication of hybrid nanocolloids via the self-assembly of polymer blends and inorganic nanoparticles.^39^ These studies collectively demonstrate that such generic models can serve as powerful tools for probing multicomponent interactions across diverse chemical contexts.

Our investigations reveal distinct morphological transformations, such as transitions from core–shell structures to dewetted states, which we quantitatively rationalize through theoretical calculations of the relative molecular interaction strengths. Furthermore, we explore the influence of stoichiometry on these morphological transitions, identifying a “rewetting” phenomenon. Finally, we extend our analysis to examine the impact of small molecules on the morphology of mixed two-component condensates. Our findings offer a molecular-level perspective for understanding and potentially controlling the morphology of multicomponent condensates through the strategic incorporation of small-molecule cosolutes.

## Molecular Model and Simulation Methodology

We employed a coarse-grained polymer model, where each monomer is represented by a single bead, with a diameter σ = 5 Å and a mass *m* = 100 g/mol, and solvent effects are treated implicitly through effective monomer–monomer pair interactions^40^ (Figure 1a). The system consists of two homopolymer species, chain A and chain B, each comprising 50 monomers of type A or B, respectively (*N* = 50). The small molecule was modeled as a single bead (type C), of the same size and mass as the polymer monomers.

**Figure 1:**
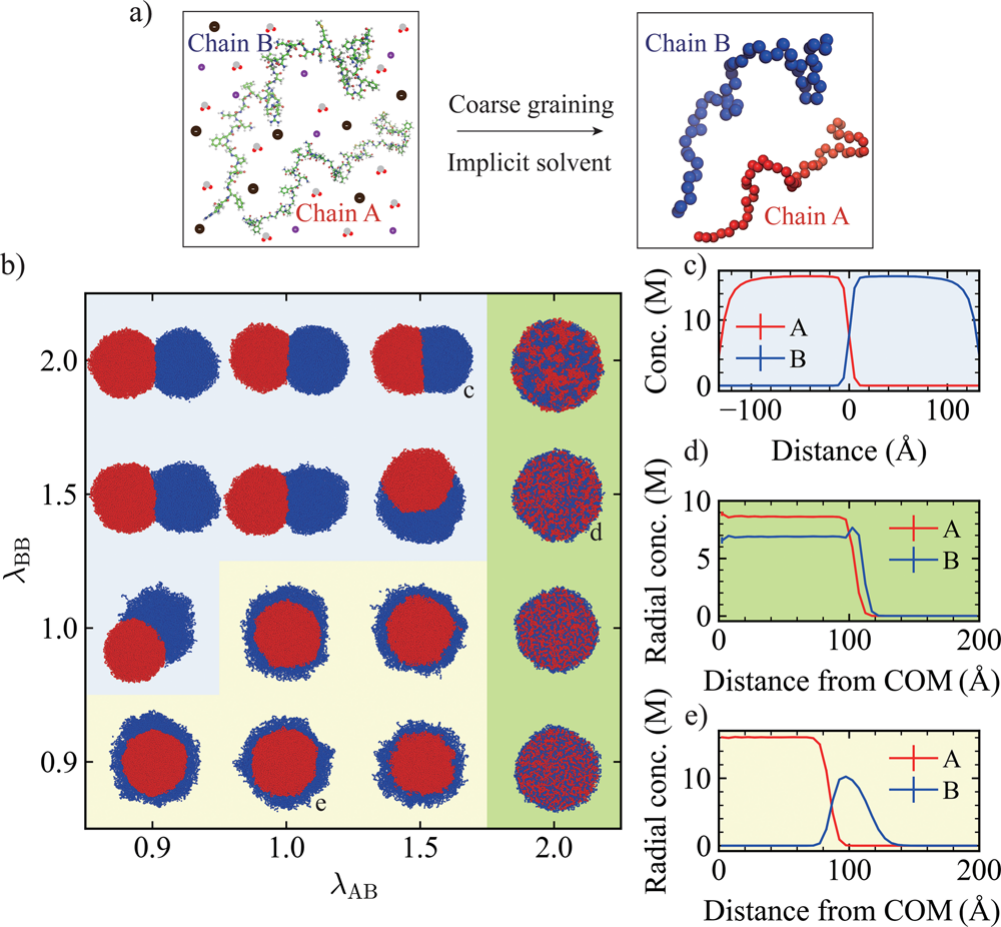
(a) Schematic of the coarse-grained polymer model in an implicit solvent. (b) Morphological state diagram of two-component condensates at various combinations of λ_AB_ and λ_BB_, with fixed λ_AA_ = 2.0. Snapshot colors correspond to the component colors shown in the schematic. (c) Concentration profiles of A and B monomers along the axis connecting the centers of mass (COM) of the A-rich and B-rich regions (λ_AB_ = 1.5, λ_BB_ = 2.0). (d,e) Radial concentration profiles of A and B monomers measured from the COM of the entire condensate for (d) λ_AB_ = 2.0, λ_BB_ = 1.5 and (e) λ_AB_ = 1.0, λ_BB_ = 0.9. Background colors indicate morphological classifications: blue for dewetted, yellow for core–shell, and green for mixed structures.

Bonded interactions between adjacent monomers were modeled using a harmonic potential

$$U_{b}\left(r\right)=\frac{k_{b}}{2}{\left(r-r_{0}\right)}^{2}$$

where *r* is the distance between bonded monomers, *k*_b_ = 20 kcal/(mol Å^2^) is the spring constant, and *r*_0_ = 3.8 Å is the equilibrium bond length.

Nonbonded interactions were described by a modified Lennard-Jones (LJ) potential, where the attractive contribution was scaled by a pairwise hydropathy parameter λ_*ij*_ between monomer types *i* and *j*

$$U_{nb}\left(r\right)=\{\begin{array}{l} U_{LJ}\left(r\right)+\left(1-\lambda_{ij}\right)\varepsilon ,,r\leq 2^{1/6}\sigma \\ \lambda_{ij}U_{LJ},,otherwise \end{array}$$

Here, *U*_LJ_(*r*) is the standard LJ potential

$$U_{LJ}\left(r\right)=4\varepsilon \left[{\left(\frac{\sigma }{r}\right)}^{12}-{\left(\frac{\sigma }{r}\right)}^{6}\right]$$

where ε = 0.2 kcal/mol is the interaction energy. By tuning the parameter λ_*ij*_, we modulate the interaction strengths between different components. Specifically, a larger λ_*ij*_ value corresponds to stronger attraction and higher hydrophobicity, while λ_*ij*_ = 0 leads to purely repulsive interactions and hydrophilic conditions.

We performed Langevin dynamics simulations using a friction coefficient γ = *m*/*t*_damp_, *t*_damp_ = 1000 ps at a constant temperature of *T* = 300 K. Simulations were run for a total of 0.5 μs with a time step of 10 fs, and the first 0.2 μs was discarded from the analysis. Error bars represent the standard error of the mean (SEM) and were computed from the final 0.3 μs, divided into 5 blocks. All simulations were conducted using HOOMD-blue (version 2.9.3) with additional features implemented via the azplugins extension (version 0.10.1). The system was simulated in a cubic box (400 Å × 400 Å × 400 Å) containing 500 A and 500 B polymers (concentration of polymer A = concentration of polymer B = 12.97 mM), which together formed a stable droplet with radius of gyration *R*_g__,droplet_ = 87.7 ± 0.006 Å, for λ_AA_ = 2.0, λ_AB_ = 1.0, λ_BB_ = 0.9. For reference, the radii of the pure 500 A and 500 B droplets were *R*_g,droplet_^pureA^ = 66.2 ± 0.002 Å and *R*_g,droplet_^pureB^ = 78.7 ± 0.01 Å, respectively, reflecting the higher hydrophobicity of the A species. Following the formation of the stable droplet, small molecules were then introduced by randomly placing additional C beads around the preformed droplet, corresponding to the target concentrations.

To quantify the effective interaction strength between the chains in the presence of small molecules, we first calculated the second virial coefficient, *B*_22_^′^, and then normalized it to obtain the dimensionless coefficient *b*_22_^′^. (The prime symbol indicates interactions in the presence of C particles.) Specifically, *b*_22_^′^ was obtained by dividing *B*_22_^′^ by its reference-state value computed in the absence of interchain attractions, thereby eliminating the influence of chain volume. *B*_22_^′^ was calculated using the following integral

$$B_{22}^{\prime }=2\pi \int_{0}^{\infty }{\left[1-e^{-\beta w\left(r\right)}\right]r}^{2}\;dr$$

where *r* is the distance between the centers of mass (COM) of two chains, *w*(*r*) is the potential of mean force (PMF) between chains, and *β* = 1/(*k*_B_ *T*). The PMF was determined via umbrella sampling simulations performed in LAMMPS using the COLVARS module. Each simulation was conducted in a cubic box of edge length 200 Å containing a single A chain and a single B chain, along with an appropriate number of C particles according to the specified concentrations (25 mM, 50 mM, 100 mM, 200 mM and 500 mM). Umbrella sampling windows were spaced every 3 Å, covering the range from 0 to 81 Å. A harmonic bias potential with a spring constant of 0.2 kcal/(mol Å^2^) was applied in each window. Each window was simulated for 100 ns using a time step of 10 fs. The first 1 ns was discarded, and observables were calculated from the remaining trajectory, divided into 5 blocks for SEM estimation.

## Result and Discussion

### Morphological Transitions in Condensates Mediated by Interaction Strength with Small-Molecule Cosolutes

To establish a reference, we initially simulated the binary system consisting of A and B chains with the solvent modeled implicitly (Figure 1a). We fixed the interactions between A–A monomer pairs to λ_AA_ = 2.0 and systematically varied the interaction strengths of B–B and A–B monomer pairs.

By adjusting the relative monomer interaction strengths between B–B and A–B, we observed distinct morphologies, including core–shell, dewetted, and mixed structures (Figure 1b). Dewetted morphologies were classified by an increased distance between the COM of the A-rich and B-rich regions, *d*_ABCOM_ (Figure S1). These structural variations were further validated using radial concentration distributions for spherical droplets and concentration gradients along the vector connecting the COM of the A-rich phase to that of the B-rich phase for nonspherical droplets (Figures 1c–e, and S2). When the A–B monomer interaction was weaker than or comparable to the B–B monomer interaction, but still weaker than the A–A monomer interaction (λ_AB_ ≤ λ_BB_ < λ_AA_), the B chains preferentially interacted with themselves rather than with the A chains, leading to the formation of a dewetted structure, as indicated by the minimal overlap in the concentration profile along the horizontal axis (Figure 1c). When λ_AB_ = λ_AA_, a mixed structure emerged (Figure 1d) and when the monomer interaction between A–B was stronger than or comparable to the B–B pairs (λ_BB_ ≤ λ_AB_ < λ_AA_), a core–shell structure formed, wherein the A chains consistently occupied the inner region (Figure 1e). This result aligns with previous findings that components with stronger homotypic interactions preferentially localize to the core.^16,39,41^ Overall, these results highlight the pivotal role of homotypic and heterotypic interactions in dictating the morphology of multicomponent condensates.

Based on the results above, we selected λ_AB_ = 1.0 with λ_BB_ = 0.9 as the initial condition, which formed a stable core–shell structure. We then investigated the effect of introducing small molecules on the condensate’s morphology. The small molecule was modeled as a single bead (C), with only excluded volume interactions between C particles (λ_CC_ = 0). Specifically, C particles were introduced at a concentration of 100 mM, initially positioned around the preformed core–shell droplet. Subsequently, the interactions of C with monomers A and B were systematically varied. To assess structural changes, we analyzed the COM distance between the phases formed by A and B chains (*d*_ABCOM_), along with configuration snapshots (cross-sectional view). In a core–shell structure, the core-forming component A and the shell-forming component B occupy distinct spatial regions within the condensate with a small *d*_ABCOM_. Conversely, a transition toward a dewetted morphology is indicated by an increase in *d*_ABCOM_, reflecting greater spatial segregation between A-rich and B-rich phases. For certain A–C and B–C interactions, we observed that the core–shell structure gradually transitioned into a dewetted morphology (Figure 2a­(i),(ii),(iv),(v)). When λ_AC_ = 0 was held constant, increasing λ_BC_ resulted in a greater partitioning ratio of C (defined as the fraction of C particles located inside the condensate, shown by green pentagons, right *y*-axis). To quantify the geometric relationship between A-rich and B-rich phases in dewetted morphologies, we calculated the intersection angle, defined in the schematic of Figure 2b as the angle between vectors connecting the intersection point to the COM of each phase (see details in Supporting Information). Correspondingly, the intersection angle emerged and gradually increased from an initially undefined state, indicating a transition from a core–shell structure to dewetting, with the degree of dewetting increasing progressively (Figure 2b). When λ_BC_ = 0 was fixed and λ_AC_ was increased, more C particles entered the condensate, consistent with recent findings that particle partitioning into biomolecular condensates is governed by condensate–particle interaction strength, even though the condensates were covered by a layer of nonattractive B particles (λ_BC_ = 0).^42^ Interestingly, although the partition ratio increased with λ_AC_, a progressive dewetting trend was not observed. Instead, dewetting occurred abruptly and only at λ_AC_ = 3.0 (Figure 2c), suggesting that, in addition to the interaction strength with the small molecules, other factors also contribute to the observed morphological transitions, a point we will return to later.

**Figure 2:**
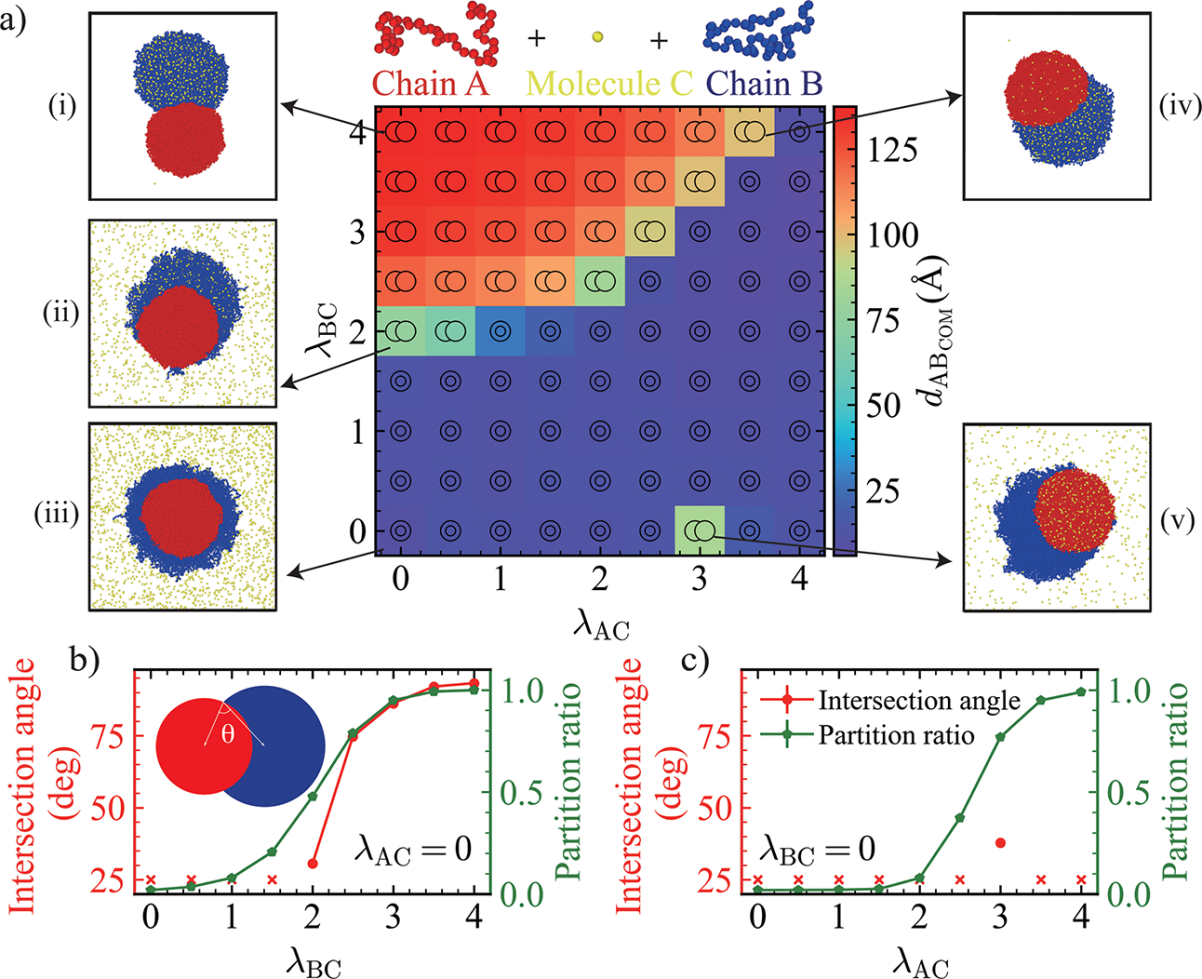
(a) Distance between the COM of the phases formed by the A and B chains, *d*_ABCOM_, as a function of λ_BC_ and λ_AC_. Colors from purple to red indicate increasing *d*_ABCOM_. Representative cross-sectional snapshots (i–v) illustrate different regimes, with colors corresponding to the schematic component colors. (b) Intersection angle (red circles, left *y*-axis) and partition ratio of C particles (green pentagons, right *y*-axis) as functions of λ_BC_ at λ_AC_ = 0. The schematic illustrates the definition of the intersection angle. (c) Intersection angle (red circles, left *y*-axis) and partition ratio (green pentagons, right *y*-axis) as functions of λ_AC_ at λ_BC_ = 0. Crosses indicate cases where no intersection angle could be defined. Concentration of C equals 100 mM.

A possible explanation for the observed morphological transitions is that the small molecules alter the effective interaction strengths among the macromolecular components of the condensate. To quantify these changes, we computed the normalized chain–chain second virial coefficient, *b*_22_^′^, in the presence of C particles. A positive *b*_22_^′^ indicates effective repulsion, while negative *b*_22_^′^ reflects effective attraction. Since A–A chain interactions are significantly stronger (with substantially more negative *b*_22_^′^) than other pairs, the A chains consistently form the inner phase in the core–shell morphologies. Therefore, to understand the morphological transition between core–shell and dewetted states, we focused primarily on the A–B and B–B chain interactions.

For purely repulsive interactions between small molecules and chains (λ_AC_ = 0, λ_BC_ = 0), the value of ${-b}_{22}^{{AB}^{\prime }}$ exceeds that of ${-b}_{22}^{{BB}^{\prime }}$ , indicating that a B chain prefers to associate with an A chain over itself, which is consistent with the monomer–monomer interactions (λ_AB_ = 1.0 > λ_BB_ = 0.9), to gain more enthalpy, resulting in a core–shell structure. However, as λ_BC_ is increased (with λ_AC_ = 0; Figure 3a), ${-b}_{22}^{{BB}^{\prime }}$ (green pentagons) increases significantly, reflecting stronger B–B interactions mediated by C particles, while ${-b}_{22}^{{AB}^{\prime }}$ (red circles) decreases due to the purely repulsive A–C interactions. At λ_BC_ = 2.0, ${-b}_{22}^{{BB}^{\prime }}$ surpasses ${-b}_{22}^{{AB}^{\prime }}$ , indicating that the B chains now favor homotypic interactions over heterotypic interactions, which is consistent with the dewetting observed in the condensate simulations. When λ_AC_ is increased at fixed λ_BC_ = 0 (Figure 3b), the B–B chain interaction remains unchanged, while the A–B chain interactions weaken with increasing λ_AC_. As shown previously, when A–B chain interactions dominate over B–B, core–shell structures are favored. However, when λ_AC_ ≥ 3.0, the B–B chain interaction becomes effectively stronger. Based on these two-chain calculations, dewetting would be expected in the multichain condensate simulations; yet, we only observe dewetting at λ_AC_ = 3.0. This mismatch between simulation results and *b*_22_^′^ ratio-based predictions suggests that more factors than the interaction strength determine condensate morphology, a point we explore in detail in the following section.

**Figure 3:**
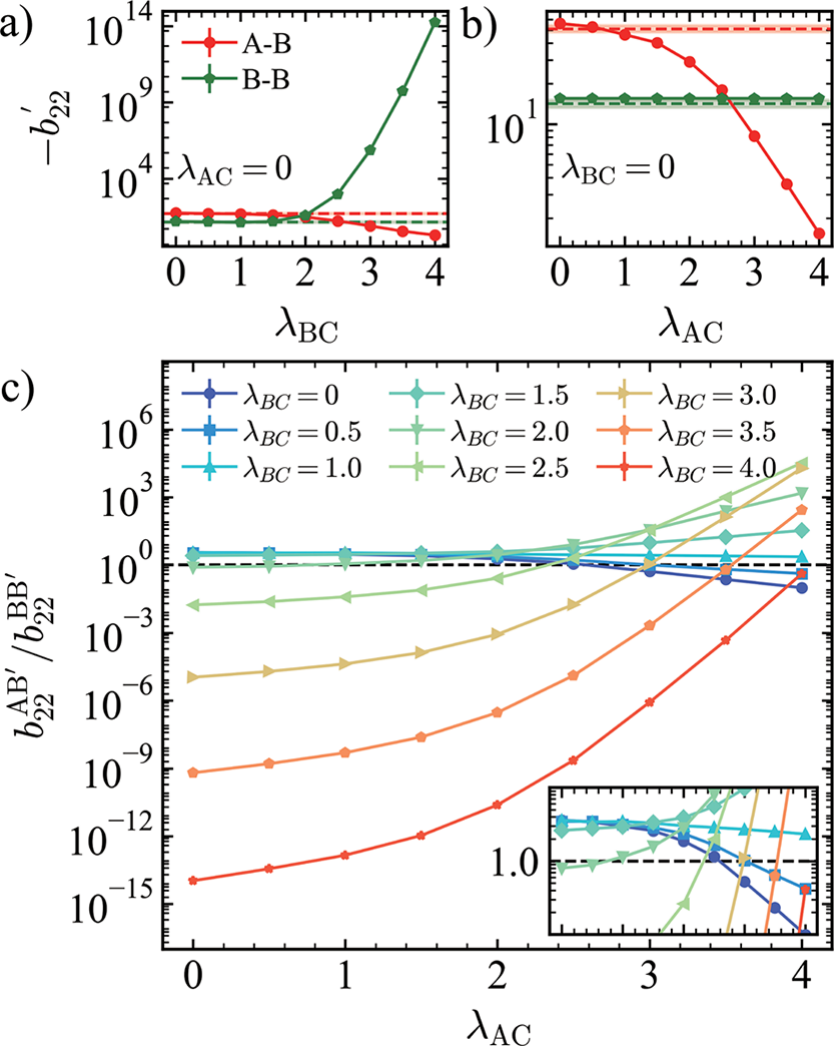
(a) Normalized chain–chain second virial coefficients (−*b*_22_^′^) for chain A–chain B (red circles) and chain B–chain B (green pentagons) as functions of λ_BC_ at λ_AC_ = 0. Red and green horizontal dashed lines represent the corresponding −*b*_22_ values in the absence of C particles. The shaded regions around each dashed line indicate the associated error bars. (b) −*b'*_22_ values for chain A–chain B (red circles) and chain B–chain B (green pentagons) as functions of λ_AC_ at λ_BC_ = 0. Horizontal dashed lines and shaded error regions are defined as in (a). (c) Ratio $b_{22}^{{AB}^{\prime }}/b_{22}^{{BB}^{\prime }}$ as a function of λ_AC_ for various λ_BC_, with color from purple to red indicating increasing λ_BC_. The inset shows a zoomed-in view of the region around a ratio of 1, using the same *x*-axis range as the main plot.

Despite these differences, the ratio $b_{22}^{{AB}^{\prime }}/b_{22}^{{BB}^{\prime }}$ generally serves as a useful metric for capturing the relative strength of A–B versus B–B chain interactions and for explaining the observed morphological transitions (Figures 3c, and S3). When the ratio exceeds one, A–B chain interactions are stronger, leading to the core–shell structure to maximize heterotypic contacts. Conversely, when the ratio falls below one, B–B chain interactions dominate, driving B chains to self-associate, and resulting in dewetting. Additionally, a greater difference between $b_{22}^{{AB}^{\prime }}$ and $b_{22}^{{BB}^{\prime }}$ corresponds to a more pronounced dewetting behavior, consistent with changes in *d*_ABCOM_.

### Stoichiometry-Dependent Modulation of Condensate Morphology by Small Molecules

While most of the observed condensate morphologies across various combinations of λ_AC_ and λ_BC_ show strong agreement with our two-chain theoretical calculations, a few notable discrepancies were identified. These inconsistencies primarily occurred at high values of λ_AC_ or λ_BC_ (≥3) and were associated with high partition ratios (Figure S4), meaning that nearly all C particles were absorbed into the condensates.

We attribute these deviations to a mismatch between the dilute conditions for chains assumed in our theoretical calculations and the high-density environment of the condensate simulations. Although the concentration of the C particles was held constant across systems, the stoichiometric ratio between the small molecules and the macromolecules varied. Compared to our two-chain calculations, the condensate simulations involve a lower stoichiometric ratio, making fewer small molecules available to mediate effective interactions between chains. In condensate simulations with strong small molecule–macromolecule interactions, the C particles fully partition into the condensate, but their number is insufficient to substantially modulate the interaction among A and B chains. As a result, even in regimes where the effective interaction strengths determined from our two-chain calculations predict dewetting, the condensates exhibit a core–shell morphology instead. These observations suggest that stoichiometry, the ratio of small molecules to chains, plays a critical role in determining condensate morphology. Motivated by this insight, we systematically varied the concentration of C particles while keeping the concentrations of A and B chains constant.

We set λ_BC_ = 0 and systematically varied the interaction strength between C particles and A chains (λ_AC_) at different concentrations of C (Figure 4a). When λ_AC_ = 0 and 1.0, the interaction between C and A monomers is weaker than the homotypic A–A monomer interaction (λ_AA_ = 2.0), and thus the condensates maintain their original core–shell morphology. However, when λ_AC_ ≥ 2.0, a clear concentration-dependent morphological transition is observed. At low concentrations of C, the condensates still retain their core–shell structure, but dewetting occurs as the concentration increases beyond a threshold. As the A–C attraction strength is increased further to λ_AC_ = 4.0, the condensate splits into orthogonal phases, one composed of B chains and one consisting of A chains and C molecules. To gain molecular-level insight into this behavior, we calculated the PMF between chain A–chain B and chain B–chain B for λ_AC_ = λ_AA_ = 2.0, which ensures that the A–A interactions remain less affected. As the concentration of component C increases, more C particles interact with chain A, effectively reducing the availability of A to interact with B. This competitive binding weakens the A–B attraction (Figure 4b). Meanwhile, although less pronounced, B–B interactions are slightly enhanced due to the excluded volume effect of C (Figure 4c). As a result, once the A–B chain interaction becomes weaker than the B–B chain interaction, dewetting is observed (Figure 4d). When the concentration of component C reaches 200 mM, *b*_22_^′^ values for the A–B and B–B chains become comparable, corresponding to a low degree of dewetting observed in the simulations. These results demonstrate that the stoichiometry between small-molecule cosolutes and condensate can significantly influence the effective interactions among condensate components, thereby driving morphology changes.

**Figure 4:**
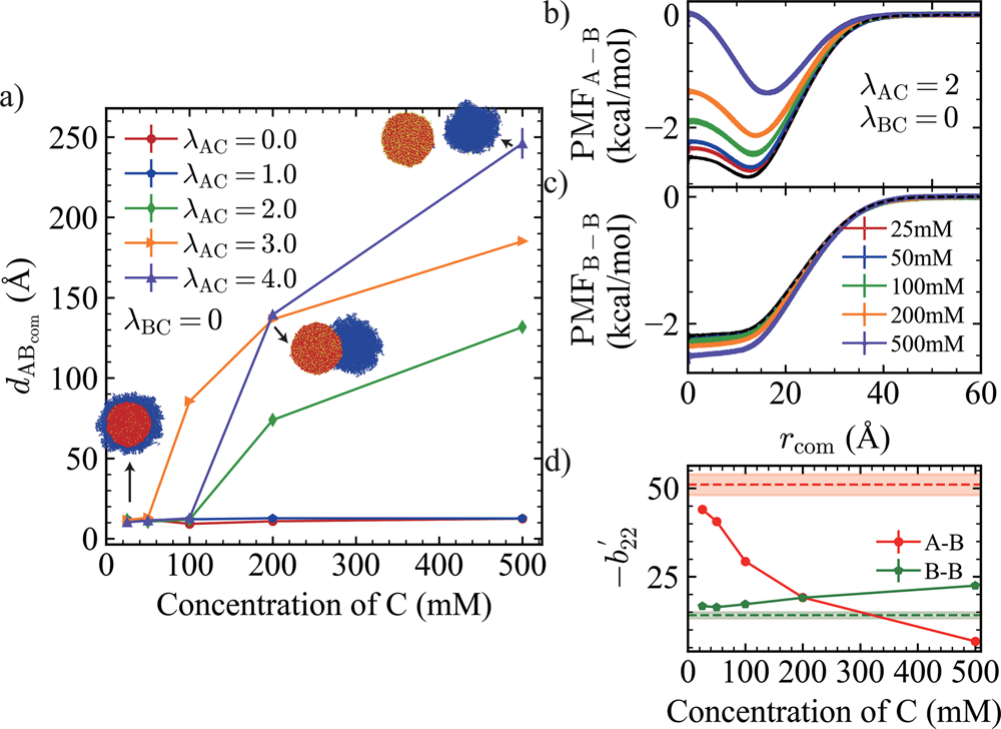
(a) *d*_ABCOM_ as a function of the concentration of component C under λ_BC_ = 0, with varying λ_AC_. Snapshots illustrate the condensate morphology for the condition λ_BC_ = 0, λ_AC_ = 4.0. (b-c) Potential of mean force (PMF) between (b) chain A and chain B, and between (c) chain B and chain B, as functions of the concentration of component C, for fixed λ_BC_ = 0, λ_AC_ = 2.0. The black dashed line indicates the PMF in the absence of particle C. (d) Normalized chain–chain second virial coefficients (−*b*_22_^′^) for chain A–chain B (red circles) and chain B–chain B (green pentagons) as functions of concentration of C at λ_BC_ = 0, λ_AC_ = 2.0. Red and green horizontal dashed lines represent the corresponding −*b*_22_ values in the absence of C particles. The shaded regions around each dashed line indicate the associated error bars.

Based on the previous results, we further varied λ_BC_ while keeping λ_AC_ = 2.0 to systematically investigate how the interaction strength and stoichiometry between the condensate components and the small molecules jointly influence morphological transitions. At low concentration of C, consistent with earlier observations, increasing λ_BC_ initially had little effect on morphology; *d*_ABCOM_ remained relatively constant. However, beyond a threshold value, *d*_ABCOM_ increased gradually, indicating a transition from a core–shell structure to a dewetted morphology (Figure 5a). At higher C concentrations (200 mM and 500 mM), we observed a nonmonotonic trend, a “re-dewetting” phenomenon, where dewetting initially appeared at low λ_BC_, then disappeared, and reemerged at higher λ_BC_ values (Figure 5a). To understand this behavior, we analyzed the normalized second virial coefficients, *b*_22_^′^, for both A–B and B–B chain pairs.

**Figure 5:**
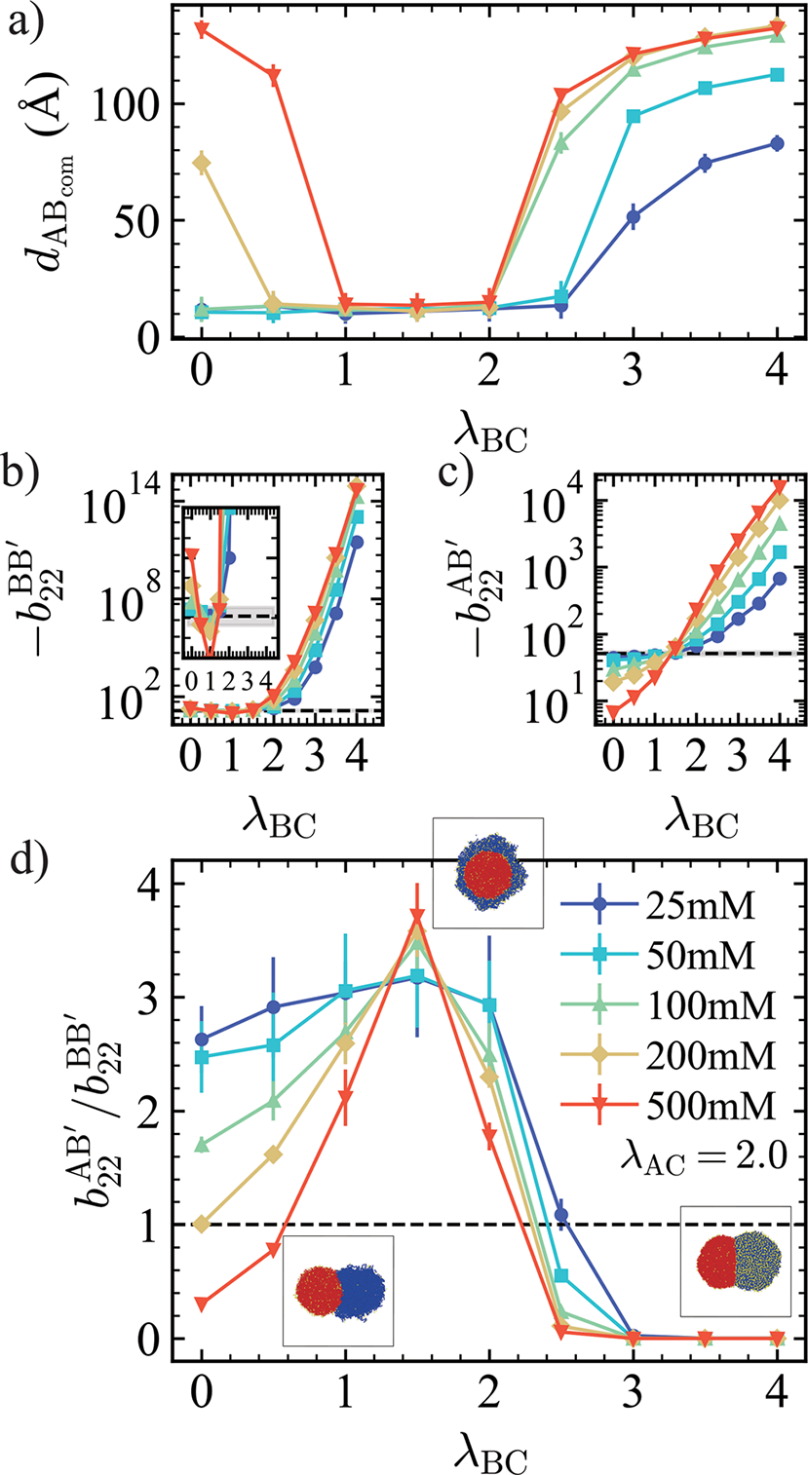
(a) *d*_ABCOM_ as a function of λ_BC_ under different concentrations of C at λ_AC_ = 2.0. (b,c) *b*_22_^′^ values for (b) chain B–chain B and (c) chain A–chain B as a function of λ_BC_ under different concentrations of C. Dashed lines represent the corresponding *b*_22_ in the absence of C. The inset in panel (b) provides a magnified view of values near the dashed line. (d) Ratio $b_{22}^{{AB}^{\prime }}/b_{22}^{{BB}^{\prime }}$ as a function of λ_BC_ for various concentrations of C. Snapshots illustrate the condensate morphologies at C = 500 mM for λ_BC_ = 0, 1.5, 4.0, respectively. All plots use the same color and marker legend as shown in panel (d).

When λ_BC_ = 0, due to volume exclusion between C–C and B–C pairs, the C particles occupy space, slightly enhancing B–B chain interactions compared to the no-C condition. As λ_BC_ increases, the effective B–B chain interaction initially decreases slightly (Figure 5b, inset), which can be attributed to the relatively weak B–C attraction leading to the weaker volume exclusion effect and disrupting B–B interaction. However, upon further increasing λ_BC_, the stronger B–C interactions begin to promote attraction between B chains. This trend becomes more pronounced at higher concentrations of C. Although we did not directly modify A–B interactions, the presence of C also affects the effective interaction between chain A and chain B as a mediator. At λ_BC_ = 0, the A–B chain interaction is weaker than in the absence of C (Figure 5c), because a portion of A interacts with C (λ_AC_ = 2.0), which is stronger than the A–B interaction. This competition weakens A–B interactions, and the effect is more pronounced at higher concentrations of C. However, as λ_BC_ increases, C can now interact with both A and B, indirectly enhancing the effective A–B interaction. This compensation effect becomes stronger as λ_BC_ increases further. Eventually, the effective A–B chain interaction exceeds the value observed in the absence of C, as more C particles bridge A and B interactions.

We continue to use the ratio $b_{22}^{{AB}^{\prime }}/b_{22}^{{BB}^{\prime }}$ as a criterion which can be either greater or less than 1, corresponding to core–shell and dewetted morphologies, respectively (Figure 5d). In the first dewetting regime at low λ_BC_, a high concentration of C enhances B–B chain interactions while weakening A–B chain interactions relative to the no-C system. Once the attraction between B–B pairs is stronger than for A–B pairs, dewetting occurs. The degree of this transition depends strongly on the concentration of C. As λ_BC_ continues to increase, the enhancement of A–B chain interactions becomes more pronounced than that of B–B, raising the ratio $b_{22}^{{AB}^{\prime }}/b_{22}^{{BB}^{\prime }}$ above 1 and resulting in a core–shell morphology. At even higher λ_BC_, B–B chain attraction once again surpasses that of A–B pairs, causing the reemergence of dewetting-a “second dewetting”. To further examine this behavior, we performed simulations scanning λ_AC_ and λ_BC_ at a C concentration of 500 mM (Figure S5). Two distinct dewetting regions were identified, and a fully separated morphology was also observed (Figures S5, and 4a).

Overall, our investigations demonstrate that the small-molecule cosolute modulates condensate morphology through its interactions with the constituent chains. This regulation is governed by both the interaction strength and stoichiometry between the small molecule and condensate components, which together determine the morphology of the condensate.

### Small Molecule-Induced Morphological Transitions in Mixed Multicomponent Condensates

As demonstrated in the previous sections, the morphology of multiphase condensates can be influenced by both the interaction strength and stoichiometry with the small molecules. However, many biologically relevant condensates are multicomponent, yet exhibit a single-phase (mixed) morphology. Therefore, we investigated the impact of small molecules on fully mixed condensates by fixing λ_AA_ = λ_AB_ = λ_BB_ = 1.0 at the 100 mM concentration of C particles. We then systematically varied the interaction strengths between C and A, B monomers, and observed the emergence of distinct morphologies (Figures 6, and S6). Specifically, when λ_AC_ = 3.0, increasing λ_BC_ led to a gradual decrease in *d*_ABCOM_. Combined with the corresponding snapshots, this trend revealed a sequence of morphological transitions: from a dewetted structure, to a core–shell structure with A chains occupying the core, to a fully mixed state, and finally to an inverse core–shell structure with B chains occupying the core (Figure 6a). To explain these transitions, we again employed the *b*_22_^′^ as a metric for the effective chain–chain interaction strengths (Figures 6b, and S7). Unlike previous cases, the A–A interaction is no longer dominant, so both $b_{22}^{{AB}^{\prime }}/b_{22}^{{AA}^{\prime }}$ and $b_{22}^{{AB}^{\prime }}/b_{22}^{{BB}^{\prime }}$ must be considered. When λ_BC_ is small, both ratios are less than 1, indicating that A–B chain interactions are weaker than the corresponding homotypic interactions (i.e., $-b_{22}^{{AA}^{\prime }}>{-b}_{22}^{{BB}^{\prime }}>-b_{22}^{{AB}^{\prime }}$ ). As a result, a dewetted structure is observed. As λ_BC_ increases, the ratio $b_{22}^{{AB}^{\prime }}/b_{22}^{{BB}^{\prime }}$ surpasses 1, while $b_{22}^{{AB}^{\prime }}/b_{22}^{{AA}^{\prime }}$ remains below 1, which implies that A–B chain interactions become stronger than B–B, while A–A interactions remain dominant, leading to the formation of a core–shell structure with A chains occupying the inner phase. Notably, at λ_BC_ = 1.5, we observe a discrepancy between the condensate simulations and the theoretical prediction based on the effective chain–chain interactions, which we attribute to stoichiometry effects, as previously discussed. Upon further increasing λ_BC_, all three interaction strengths approach equivalence, i.e., $-b_{22}^{{AA}^{\prime }}\approx {-b}_{22}^{{BB}^{\prime }}\approx -b_{22}^{{AB}^{\prime }}$ , resulting in a fully mixed structure. If λ_BC_ continues to increase, B–B chain interactions eventually become the strongest, leading to the formation of an inverse core–shell structure with B chains occupying the interior. These results demonstrate that in a single-phase multicomponent system, the small molecules can effectively regulate condensate morphology by altering the relative effective interaction strengths among the constituent chains.

**Figure 6:**
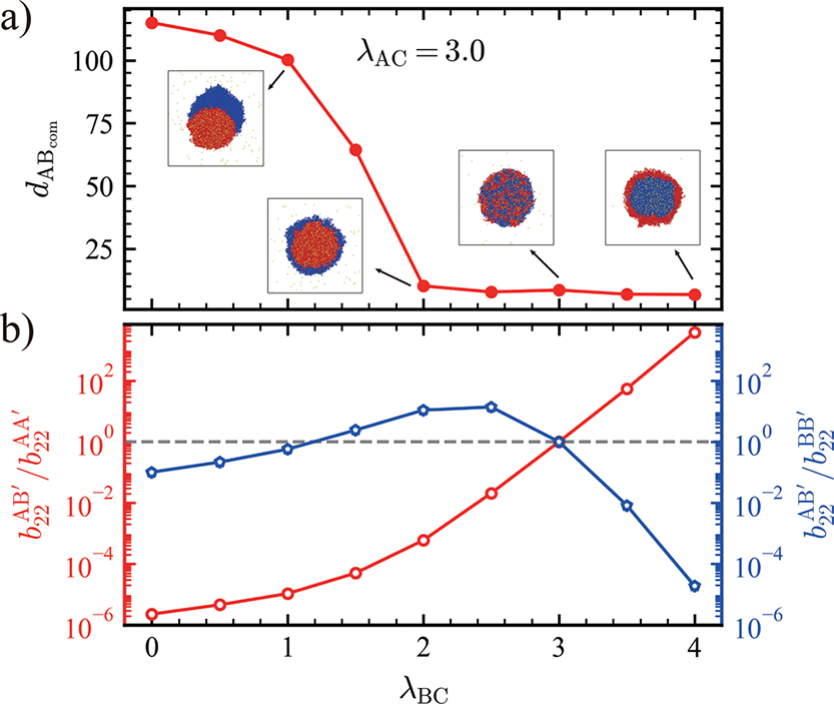
(a) *d*_ABCOM_ as a function of λ_BC_ at fixed λ_AC_ = 3.0. Snapshots show cross-sectional views of representative condensate morphologies. (b) Ratio $b_{22}^{{AB}^{\prime }}/b_{22}^{{AA}^{\prime }}$ (left *y*-axis, red) and ratio $b_{22}^{{AB}^{\prime }}/b_{22}^{{BB}^{\prime }}$ (right *y*-axis, blue) as a function of λ_BC_ under λ_AC_ = 3.0.

## Conclusions

In this study, we investigated how small-molecule cosolutes modulate the morphology of multicomponent condensates from a molecular interaction perspective. By systematically varying the interaction strengths between the small molecules and each condensate component, we demonstrated that small molecules can effectively reshape the condensate morphology. To interpret these transitions, we computed normalized effective second virial coefficients, *b*_22_^′^, to quantify homotypic and heterotypic interactions between two chains in the presence of small molecules, and demonstrated that the morphological changes arise from shifts in their relative strengths.

By systematically varying the concentration of the small molecules, we further showed that concentration-dependent modulation of effective interactions can account for highly nonlinear morphological transitions observed in simulations. We also extended our analysis to multicomponent condensates that are fully mixed in the absence of cosolutes, and found that tuning the relative interaction strengths through small molecules could induce transitions to different morphologies such as dewetted, core–shell, or inverse core–shell.

This molecular interaction-based framework can also be extended to macromolecular regulators, such as RNAs and proteins, which have similarly been shown to influence condensate morphology.^18,19^ Although these macromolecules often exhibit multivalency, the resulting morphological transitions in multicomponent condensates still arise from the same fundamental principle: the balance between effective homotypic and heterotypic interactions. Whether mediated by small-molecule cosolutes or larger biomolecules^43^ such as proteins and nucleic acids, additives modulate these interactions through their differential affinities for the condensate components and their stoichiometric ratios relative to the macromolecules (Figure 7). When a sufficient number of additive molecules are present, asymmetric affinities can shift the balance between homotypic and heterotypic interactions, thereby driving changes in condensate morphology. Recent experiments have highlighted the capacity of additives to modulate condensate organization. For example, Zhu et al. demonstrated that Hofmeister series ions can either enhance or weaken the effective homotypic interactions between proteins, thereby driving transitions between miscible and immiscible states.^26^ In a study by Ye et al., the introduction of RNA into an initially mixed condensate reduced the effective heterotypic interactions by strongly binding to one of the components. This interaction led to a morphological transition in which the other component became enriched at the interface, and further increasing the RNA concentration amplified this interfacial enrichment.^44^ In a more complex ternary system studied by Lu et al., which forms a core–shell structure, the introduction of α-synuclein, which has a stronger affinity for one component in one phase, weakened the effective interaction mediated by the shared component between the two phases. This reduction in interphase interactions promoted the formation of a dewetted morphology.^45^ Together, these examples illustrate how modulation of homotypic and heterotypic interactions by additives controls condensate morphology. Our findings provide a mechanistic framework for understanding and engineering such morphological transitions, offering insights that may inform future experimental and computational efforts to control condensate structure and function across diverse biological contexts.

**Figure 7:**
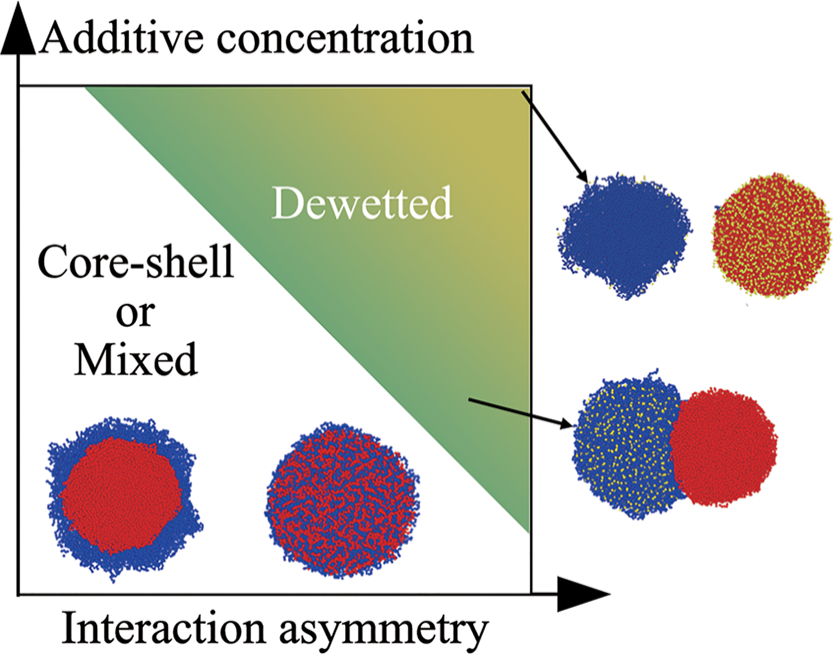
Schematic phase diagram illustrating the dependence of condensate morphology on additive concentration and the interaction asymmetry between the additive and two condensate components. Dewetting occurs when either λ_AC_ > λ_BC_ or λ_AC_ < λ_BC_.

## Supplementary Material



## Notes

Descriptions of the intersection angle calculation method and density profile analysis; density profiles; normalized second virial coefficients between components under various conditions; partition ratio diagrams; state diagrams characterized by center-of-mass distance; summary tables of all simulated parameters and results (PDF)
