# Supplementary material for: Controlling Multicomponent Condensate Morphology via Additive-Modulated Interactions
Source: JACS Au. 2025 Jul 30;5(8):4064–72. doi: 10.1021/jacsau.5c00713 (PMC12381699; doi:10.1021/jacsau.5c00713)
Supplement: Supplementary file 1 [file au5c00713_si_001.pdf]

Supporting Information for Publication

Controlling Multicomponent Condensate Morphology via Additive-Modulated Interactions

Jiahui Wang,[1,*] Arash Nikoubashman,[2,3,4] Young C. Kim[5] and Jeetain Mittal[1,6,7,*]

[1]Artie McFerrin Department of Chemical Engineering, Texas A&M University, College Station, TX 77843, United States

[2]Leibniz-Institut für Polymerforschung Dresden e.V., Hohe Straße 6, 01069 Dresden, Germany

[3]Institut für Theoretische Physik, Technische Universität Dresden, 01069 Dresden, Germany

[4]Cluster of Excellence Physics of Life, Technische Universität Dresden, 01062 Dresden, Germany

[5]Center for Materials Physics and Technology, Naval Research Laboratory, Washington, DC 20375, United States

[6]Department of Chemistry, Texas A&M University, College Station, TX 77843, United States

[7]Interdisciplinary Graduate Program in Genetics and Genomics, Texas A&M University, College Station, TX 77843, United States

[*]Corresponding author email:

jhwang@tamu.edu

jeetain@tamu.edu

Analysis Methods

Intersection Angle Calculation

To quantify the geometric relationship between A-rich and B-rich phases in dewetted morphologies, we first defined the axis connecting their respective centers of mass (COMs) as the y-axis. An orthogonal direction was designated as the x-axis, forming an x-y plane onto which all particle coordinates were projected. This plane was discretized into a 200×200 grid. For each simulation frame, number density maps were generated on this grid, and a contour line was extracted at 90% of the maximum density to represent the phase boundary. Each contour was fitted to a circle, and the intersection points of the two circles were identified. The intersection angle was then defined as the angle between the lines connecting each COM to the intersection point.

Density Profile Along the Horizontal Axis

To evaluate the spatial distribution of components in dewetted morphologies, we used the vector connecting the COMs of the A-rich and B-rich phases as the horizontal axis. The system was divided into 50 bins along this direction. The concentrations of A and B monomers were calculated in each bin to construct density profiles.

Figures

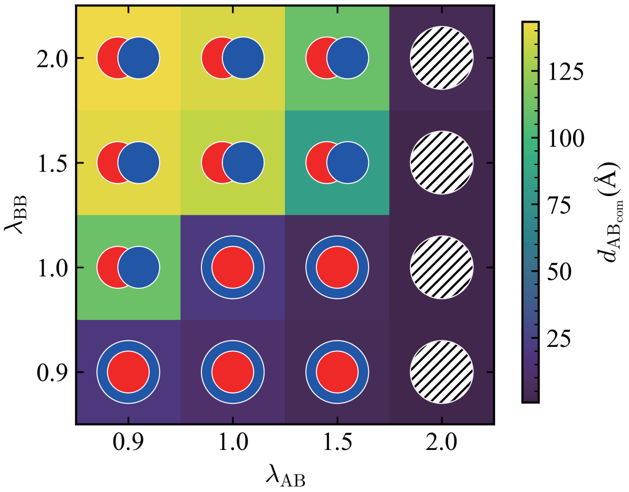

Fig. S1 Distance between the center of mass (COM) of the phases formed by the A and B chains, $d_{AB_{COM}}$, as a function of λ_{AB} and λ_{BB}. Colors from dark purple to yellow indicate increasing $d_{AB_{COM}}$. The schematic representations illustrate the corresponding morphologies: two concentric circles indicate a core-shell structure, two adjacent circles represent a dewetted morphology, and a single hatched circle denotes a mixed structure.

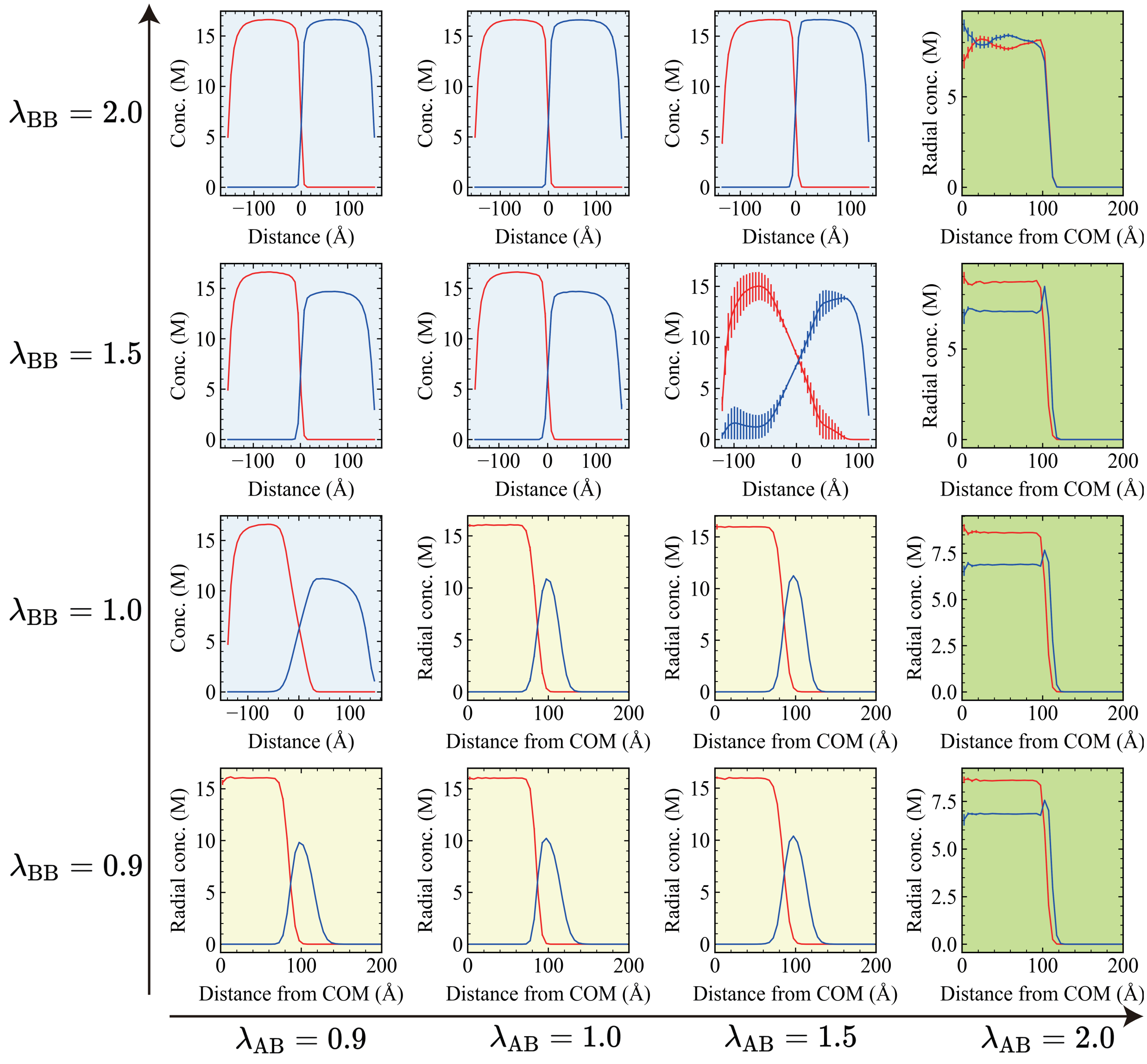

Fig.S2 (a) Concentration profiles of A (red line) and B (blue line) monomers under different interaction strengths. Radial concentration distributions for spherical droplets and concentration gradients along the vector connecting the center of mass (COM) of the A-rich phase to that of the B-rich phase for non-spherical droplets. Background colors indicate morphological classifications: blue for dewetted, yellow for core-shell, and green for mixed structures.

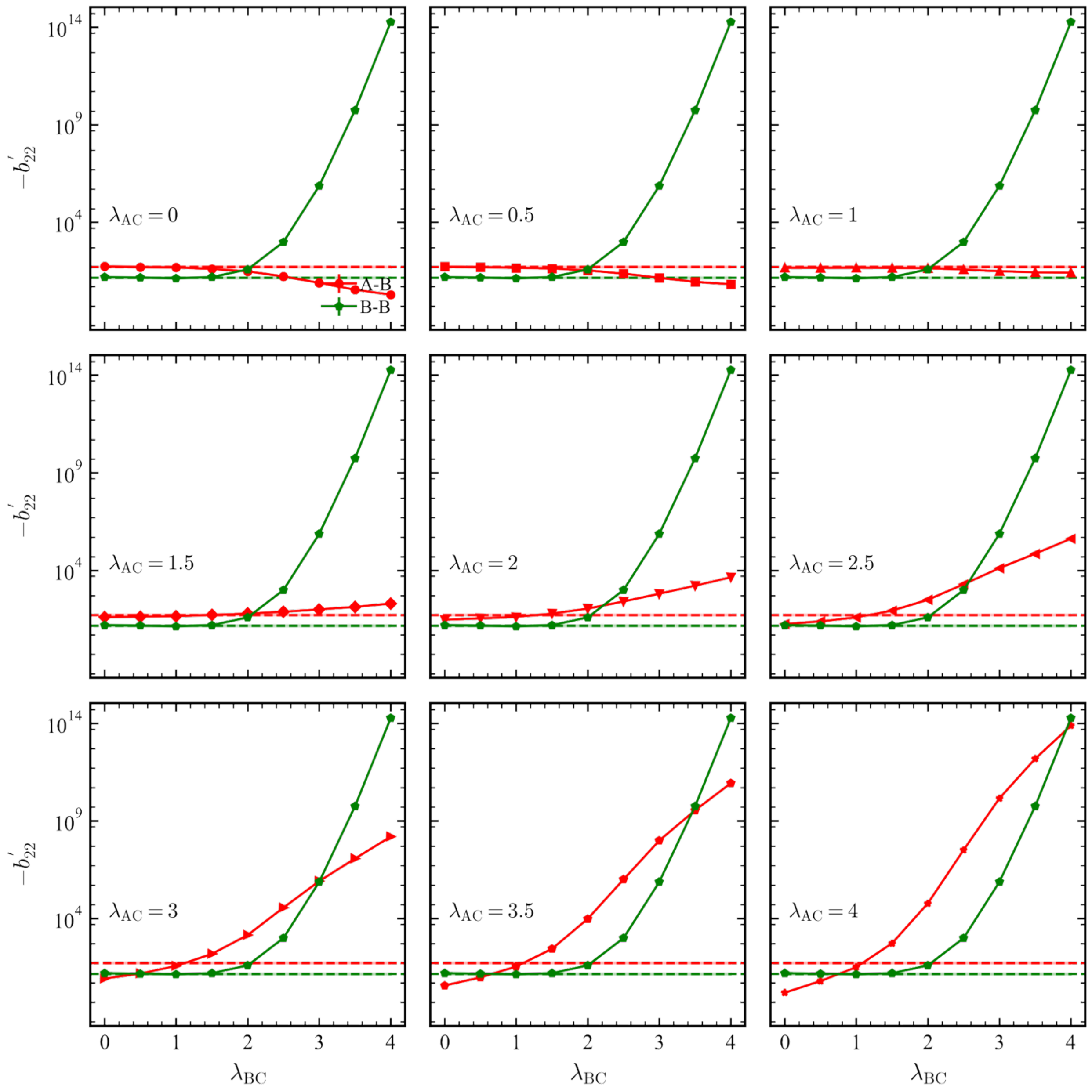

Fig.S3 $-b_{22}$ values for Chain A-ChainB (red) and Chain B-Chain B(green) as functions of λ_{BC} at varies constant λ_{AC}. Red and green horizontal dashed lines represent the corresponding $-b_{22}$ values in the absence of C particles. The shaded regions around each dashed line indicate the associated error bars.

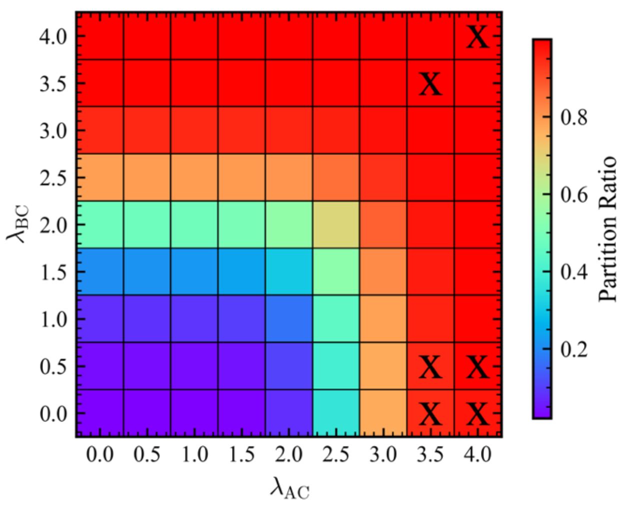

Fig.S4 Partition ratio as a function of λ_{BC} and λ_{AC}. Colors from purple to red indicate increasing partition ratios. Squares marked with an “X” represent conditions where simulation results are inconsistent with theoretical predictions.

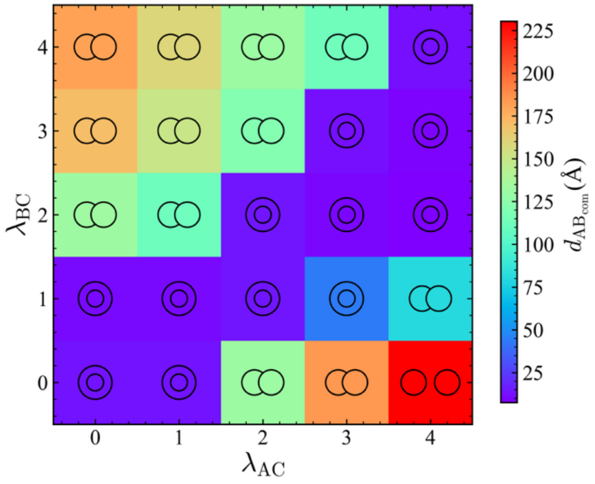

Fig. S5 Distance between the COM of the phases formed by the A and B chains, $d_{\mathrm{AB_{COM}}}$, as a function of λ_{BC} and λ_{AC}. Concentration of C equals 500 mM. The schematic representations illustrate the corresponding morphologies: two concentric circles indicate a core-shell structure, two adjacent circles represent a dewetted morphology, and two completely separated circles indicate fully demixed droplets.

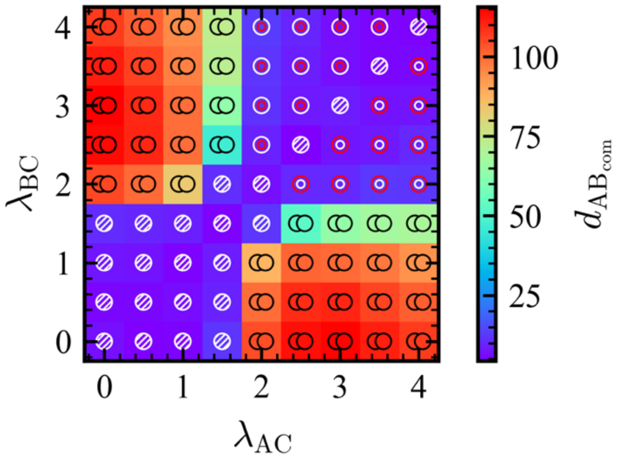

Fig.S6 (a) $d_{AB_{COM}}$, as a function of λ_{BC} and λ_{AC}. Colors from purple to red indicate increasing $d_{AB_{COM}}$. The marker on each colored square denotes the observed morphology: mixed (circle with hatch), dewetting (two connected circles), core-shell (concentric circles with red core and white shell), and inverse core-shell (concentric circles with white core and red shell).

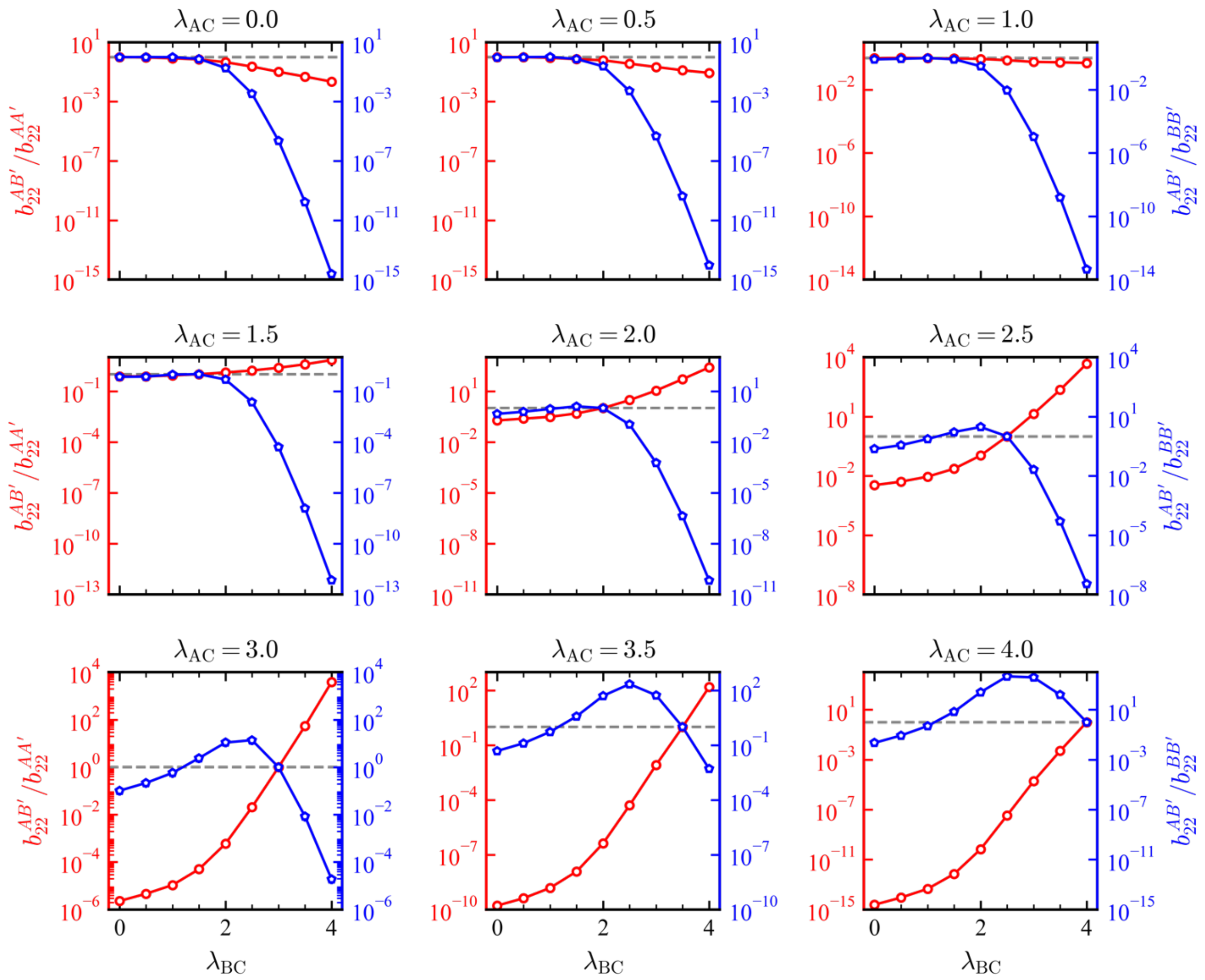

Fig. S7 Ratio $b_{22}^{\mathrm{AB}'}/b_{22}^{\mathrm{AA}'}$ (left *y*-axis, red) and ratio $b_{22}^{\mathrm{AB}'}/b_{22}^{\mathrm{BB}'}$ (right *y*-axis, blue) as a function of λ_{BC} under different λ_{AC}.

Table 1. Morphologies observed in each simulated case at an additive concentration of 100 mM, with $\lambda_{AA} = 2.0, \lambda_{AB} = 1.0, \lambda_{BB} = 0.9$.

$\boldsymbol{\lambda_{AC}}$	$\boldsymbol{\lambda_{BC}}$	**Morphology**
0.0	2.0, 2.5, 3.0, 3.5, 4.0	Dewetted
0.5	2.0, 2.5, 3.0, 3.5, 4.0	
1.0	2.5, 3.0, 3.5, 4.0	
1.5	2.5, 3.0, 3.5, 4.0	
2.0	2.5, 3.0, 3.5, 4.0	
2.5	3.0, 3.5, 4.0	
3.0	0.0, 3.5, 4.0	
3.5	4.0	
0.0	0.0, 0.5, 1.0, 1.5	Core-shell
0.5	0.0, 0.5, 1.0, 1.5	
1.0	0.0, 0.5, 1.0, 1.5,2.0	
1.5	0.0, 0.5, 1.0, 1.5, 2.0	
2.0	0.0, 0.5, 1.0, 1.5, 2.0	
2.5	0.0, 0.5, 1.0, 1.5, 2.0,2.5	
3.0	0.5, 1.0, 1.5, 2.0, 2.5, 3.0	
3.5	0.0, 0.5, 1.0, 1.5, 2.5, 3.0, 3.5	
4.0	0.0, 0.5, 1.0, 1.5, 2.0, 2.5, 3.0, 3.5 ,4.0	

Table 2. Morphologies observed in each simulated case at an additive concentration of 500 mM, with $\lambda_{AA} = 2.0, \lambda_{AB} = 1.0, \lambda_{BB} = 0.9$.

$\boldsymbol{\lambda_{AC}}$	$\boldsymbol{\lambda_{BC}}$	**Morphology**
0.0	2.0, 3.0, 4.0	
1.0	2.0, 3.0, 4.0	
2.0	0.0, 0.5, 2.5, 3.0, 3.5, 4.0	Dewetted
3.0	0.0, 4.0	
4.0	0.0, 1.0	
0.0	0.0, 1.0	
1.0	0.0, 1.0	
2.0	1.0, 1.5, 2.0	Core-shell
3.0	1.0, 2.0,3.0	
4.0	2.0,3.0,4.0	

Table 3. Morphologies observed in each simulated case at an additive concentration of 25 mM, with $\lambda_{AA} = 2.0, \lambda_{AB} = 1.0, \lambda_{BB} = 0.9$.

$\boldsymbol{\lambda_{AC}}$	$\boldsymbol{\lambda_{BC}}$	**Morphology**
2.0	3.0,3.5,4.0	Dewetted
0.0	0.0	
1.0	0.0	
2.0	0.0, 0.5,1.0, 1.5, 2.0, 2.5	Core-shell
3.0	0.0	
4.0	0.0	

Table 4. Morphologies observed in each simulated case at an additive concentration of 50 mM, with $\lambda_{\mathrm{AA}} = 2.0, \lambda_{\mathrm{AB}} = 1.0, \lambda_{\mathrm{BB}} = 0.9$.

$\boldsymbol{\lambda}_{\mathbf{AC}}$	$\boldsymbol{\lambda}_{\mathbf{BC}}$	**Morphology**
2.0	3.0,3.5,4.0	Dewetted
0.0	0.0	Core-shell
1.0	0.0	
2.0	0.0, 0.5,1.0, 1.5, 2.0, 2.5	
3.0	0.0	
4.0	0.0	

Table 5. Morphologies observed in each simulated case at an additive concentration of 200 mM, with $\lambda_{\mathrm{AA}} = 2.0, \lambda_{\mathrm{AB}} = 1.0, \lambda_{\mathrm{BB}} = 0.9$.

$\boldsymbol{\lambda}_{\mathbf{AC}}$	$\boldsymbol{\lambda}_{\mathbf{BC}}$	**Morphology**
2.0	0.0, 2.5, 3.0, 3.5, 4.0	Dewetted
3.0	0.0	
4.0	0.0	
0.0	0.0	Core-shell
1.0	0.0	
2.0	0.5, 1.0, 1.5, 2.0	

Table 6. Morphologies observed in each simulated case at an additive concentration of 100 mM, with $\lambda_{AA} = \lambda_{AB} = \lambda_{BB} = 1.0$.

$\boldsymbol{\lambda_{AC}}$	$\boldsymbol{\lambda_{BC}}$	**Morphology**
0.0	2.0, 2.5, 3.0, 3.5, 4.0	Dewetted
0.5	2.0, 2.5, 3.0, 3.5, 4.0	
1.0	2.0, 2.5, 3.0, 3.5, 4.0	
1.5	2.5, 3.0, 3.5, 4.0	
2.0	0.0, 0.5, 1.0	
2.5	0.0, 0.5, 1.0, 1.5	
3.0	0.0, 0.5, 1.0, 1.5	
3.5	0.0, 0.5, 1.0, 1.5	
4.0	0.0, 0.5, 1.0, 1.5	
2.0	2.5, 3.0, 3.5, 4.0	Core-shell (B occupy core)
2.5	3.0, 3.5, 4.0	
3.0	3.5, 4.0	
3.5	4.0	
2.5	2.0	Core-shell (A occupy core)
3.0	2.0, 2.5	
3.5	2.0, 2.5, 3.0	
4.0	2.0, 2.5, 3.0, 3.5	
0.0	0.0, 0.5, 1.0, 1.5	Mixed structure
0.5	0.0, 0.5, 1.0, 1.5	
1.0	0.0, 0.5, 1.0, 1.5	
1.5	0.0, 0.5, 1.0, 1.5, 2.0	
2.0	1.5, 2.0	
2.5	2.5	
3.0	3.0	
3.5	3.5	
4.0	4.0	
